# Decompressive Hemicraniectomy for Stroke in Older Adults: A Review

**DOI:** 10.29245/2572.942X/2017/2.942X/2017/1.1103

**Published:** 2016-11-22

**Authors:** Faith C. Robertson, Hormuzdiyar H. Dasenbrock, William B. Gormley

**Affiliations:** 1 Harvard Medical School, Boston, Massachusetts, United States of America; 2 Cushing Neurosurgical Outcomes Center, Brigham and Women’s Hospital, Boston, Massachusetts, United States of America; 3 Department of Neurological Surgery, Brigham and Women’s Hospital, Boston, Massachusetts, United States of America

**Keywords:** Age, Cerebral infarction, Decompressive craniectomy, Hemicraniectomy, Ischemic stroke, Middle cerebral artery

## Abstract

Malignant cerebral edema is a potential consequence of large territory cerebral infarction, as the resultant elevation in intracranial pressure may progress to transtentorial herniation, brainstem compression, and death. In appropriate patients, decompressive hemicraniectomy (DHC) reduces mortality without increasing the risk of severe disability. However, as the foundational DHC randomized, controlled trials excluded patients greater than 60 years of age, the appropriateness of DHC in older adults remains controversial. Recent clinical trials among elderly participants, including DESTINY II, reported that DHC reduces mortality, but may leave patients with substantial morbidity. Nationwide analyses have demonstrated generalizability of such data. However, what constitutes an acceptable outcome – the perspective on quality of life after survival with substantial disability – varies between clinicians, patients, and caregivers. Consequently, quality of life measures are being increasingly incorporated into stroke research. This review summarizes the impact of DHC in space-occupying cerebral infarction, and the influence of patient age on postoperative survival, functional capacity, and quality of life—all key factors in the clinical decision process. Ultimately, these data underscore the inherent complexity in balancing scientific evidence, clinical expertise, and patient and family preference when pursuing hemicraniectomy among the elderly.

## Introduction

Acute ischemic stroke disproportionally affects older individuals^47^ and as benefits of procedural interventions for stroke can vary by age^20^ it is imperative to understand how medical and surgical expectations change for older patients. In large vessel acute ischemic stroke, the evolution of space-occupying cerebral infarctions to malignant cerebral edema is a major cause of neurologic morbidity and mortality, as increases in intracranial pressure progress to transtentorial herniation and brainstem compression^19^. The benefit of surgical intervention was demonstrated in the last decade when three European randomized trials were conducted simultaneously to compare decompressive hemicraniectomy (DHC) to conservative management^17,21,26,37^. However, these trials excluded patients over 60 years of age, leaving to question the appropriateness of DHC for elderly patients.

As DHC utilization continues to increase in the setting of acute ischemic stroke^1,42^ realizing the impact of patient age on DHC outcomes is necessary, particularly in the setting of an increasing life expectancy for people with multiple comorbidities and stroke risk factors^12^. This review involved a literature research of PubMed to identify relevant studies published before November 2016. The search strategy included the following MeSH terms and keywords: (“hemicraniectomy” OR “decompressive surgery” OR “DHC”) AND (“age” OR “elderly” OR “older patients”) AND “stroke”. Titles and abstracts were reviewed to identify potentially relevant studies. We summarize the utility of DHC, the current understanding of how age (particularly age greater than 60 years) influences postoperative survival, functional capacity, and quality of life, and how that data impacts the complex decision analysis in the clinical setting.

## Decompressive Hemicraniectomy for Stroke

Surgical decompression became a prominent treatment option for acute ischemic stroke in the 1990s^7,19,32^. Multiple observational studies suggested that DHC provided a mortality benefit compared with medical management, for which mortality was 7-80%^4,7–10,18,22,28,30,36,43,45,48^. However, authors called for an RCT to confirm the efficacy of surgical intervention. As many institutional studies reported that age negatively impacted patient outcomes^7,10,18,22,36,48^ the initial RCTs on DHC restricted the age of trial participants^17^,^21^,^26^,^37,38^. DECIMAL was a French multicenter, randomized trial involving 38 patients between 18-55 years of age^37^. DESTINY enrolled 32 German patients aged 18-60 years^26^. HAMLET, the Dutch trial, included 64 patients aged 18-60 years^21^. The data were combined in a pooled analysis, and showed a 50% absolute risk reduction for mortality, further supporting survival benefit of DHC.

While mortality is often the primary outcome utilized in clinical trials, an individual’s functional status is of equal if not more importance, particularly to the patient and caregivers. Quality outcomes are frequently measured with the modified Rankin Scale, (mRS; Table 1). The pooled-analysis of European RCTs showed improved quality outcomes with surgery: a 23% absolute risk reduction of mRS score <3, and a 51% absolute risk reduction of mRS score <4^37^. Subgroup analysis of outcomes by age (dichotomized at 50) was underpowered^38^.

As evidence supporting DHC for stroke treatment increased, so did utilization^1,6,42^. A nationwide study of DHC use in America circa the European trials’ publication noted a 3-fold increase in DHC between 1999 and 2008^42^. Nevertheless, the question of DHC appropriateness in patients aged greater than 60 years remained.

## Use of Decompressive Hemicraniectomy in Older Patients

The understanding of DHC efficacy in older patients prior to recent RCTs was shaped by a combination of heterogeneous results from small institutional studies, which often lacked clear selection criteria and adequate representation of all age groups (Table 2)^4,7,8,10,18,22,24,30,34,36,48^. A study in 1997 was one of the first to highlight age-related differences in postoperative function^7^. Carter et al. reported five of five patients <50 years old with good postoperative mobility and self-care (Barthel Index scores >60), verses three of six older patients. In 2001, an institutional study of patients >55 years old showed that DHC decreased mortality, but all survivors had mRS scores ≥4^22^. In 2004, eight neurosurgical department databases were combined (188 patients) and showed that patients >50 years of age had significantly worse outcomes (12.0% of older patients could function independently, versus 34.9% of younger individuals)^36^. Importantly, many early institutional studies likely had unadjusted confounding from patient comorbidities, delayed surgery (intervention after >48 hours), or transtentorial herniation (clinical signs of decreased arousal, brainstem compression)^23,25,35,39^. Regardless, many authors recommended avoiding DHC in elderly patients unless a prospective randomized trial proved benefit.

The paucity of class I evidence on patients >60 years of age prompted additional trials to specifically analyze the utility of DHC among older adults^16,27,35,49,50^. In 2012, Zhao et al. published results of a prospective RCT for DHC versus conservative treatment enrolling 47 patients up to 80 years of age^50^. In subgroup analysis of patients older than 60, DHC decreased 6-month mortality more than conservative management (12.5% versus 61.5%, respectively). Regarding functional status, 31.2% (n=16) of DHC patients experienced poor outcome (mRS >4), versus 92.3% (n=13) of individuals allocated to the medical treatment arm. However, interpretation of this data is limited by the small sample size and fact that >60% of patients were from a single institution.

In 2014, the New England Journal of Medicine published DESTINY II, a German multicenter RCT investigating the efficacy of DHC in 112 patients >60 years of age^27^. The primary end point was survival without severe disability (mRS ≤4), 6 months after randomization. In the DHC group, 38% survived without severe disability, compared to 18% in the control arm. The proportional differences between groups were dominated by mortality rates (33% of surgical patients versus 70% medical). At 6 months, no patients had mRS scores <2; 7% of DHC patients and 3% of control patients had an mRS=3; 32% and 15%, respectively, had scores of 4; and 28% and 13%, mRS=5. When comparing 12 month data to the original DESTINY trial of patients ≤60 years of age, only 6% of older DHC patients had an mRS score <4, compared to 43% of younger counterparts. Therefore, DHC in older patients reduced mortality, but did not provide substantial gain in functional outcomes.

A subsequent meta-analysis assessing 14 studies and 747 patients greater or less than 60 years of age found that while all ages benefited from early intervention (DHC within 48 hours of stroke onset), older age was an important predictor of unfavorable outcome^28^. Patients >60 years of age had an 88.3% odds of mRS >3, which was significantly higher than younger patients (66.8%).

While RCTs provide the most internally valid form of clinical evidence for treatment efficacy, the effectiveness, or generalizability of such data to the clinical setting may be variable. Nationwide population studies offer alternative means to elucidate the degree to which RCT findings are congruent with daily practice. In our recent nationwide database analysis, we used the Nationwide Inpatient Sample, the largest all-payer inpatient health care database in the US, to determine how age impacted DHC-related morbidity and mortality^11^. The study included 1,673 patients with space-occupying cerebral infarction from hospitals in almost every American state. The large sample size, 37.6% >60 years of age, allowed us to account for confounding factors, including individual comorbidities, stroke risk factors, treatment variables, and the timing of intervention in multivariable regression analyses. We reported survival benefit of DHC compared to medical treatment in all age groups, including those >70 years. However, patients >60 years had higher odds of postoperative mortality compared with younger patients (aged 18–50 years: 19%, 51–60 years: 22.8%, 61–70 years: 30.7%, >70 years: 34.5%). As mRS scores were not coded in the database, the variables of mortality, discharge to an institutional care facility, and tracheostomy or gastrostomy placement served as proxies for mRS score and were designated as a “poor outcome”^44^. Patients aged >60 years had increased odds of discharge to institutional care (47.1%), and overall poor outcome (77.0%).

Another nationwide study using the Medicare claims database, a federally funded health insurance program for patients >65 years of age in the United States, examined older patients’ postoperative outcomes and use of long-term care facilities, as a proxy for independence^13^. Within 30 days of surgery, 38% of patients died, and 25% of survivors required acute hospital readmission. At one year, 55% of patients were deceased, and 29% of survivors required full-time care at a long-term care facility. This lies in contrast to the patients >60 years of age in the DESTINY II trial where, at one year, mortality was 43% and 57% of survivors required significant assistance (mRS 4-5)^27^.

In summary, while there is a general consensus from RCT, institutional, and nationwide data that DHC in patients >60 years of age decreases the odds of mortality compared to medical management, there remains a significant risk of morbidity for survivors. The aforementioned studies note that between 25% and 62% of patients will be left with moderately severe disability following DHC (mRS >3), and may be unable to perform activities of daily living unassisted.

## Perspective on Quality of Life

Numerous studies in the past decade have proven that DHC in older patients is lifesaving, however, the risk of survival with moderate or severe disability fuels the ongoing debate about what should be considered an acceptable outcome for these patients^4,5,14,15,33^. Importantly, the perspective on the acceptability of survival with substantial disability can vary between clinicians, patients, and caregivers. In a recent multicenter, international, cross-sectional survey among 1,860 physicians who care for stroke patients, mRS scores ≤3 were considered acceptable by the majority (79.3%), while less than half (38.0%) considered mRS =4 to be an acceptable outcome^29^. However, the patient experience of stroke recovery may be better captured by quality of life (QoL) measures^15,40,46^. QoL is traditionally measured by Short-Form Health Survey (SF-36), Hamilton Depression Rating Scale (HDRS), Stroke Impact Scale (SIS), and European QoL Scale (EQ-5D). In DESTINY II, patients who underwent DHC had higher QoL scores than those treated conservatively (via SF-36, HDRS, and EQ-5D).

As QoL data are not routinely collected as primary outcomes in RCTs, Ali et al. examined the correlation of patients’ perspectives on QoL with traditional functional outcome measures: mRS score, National Institutes of Health Stroke Scale, and Barthel Index^3^. QoL was assessed with the SIS and EQ-5D. The mRS aligned most with stroke survivors’ interests, capturing more information on QoL than either the National Institutes of Health Stroke Scale or Barthel Index.

Retrospective consent is another approach to understanding patients’ and caregivers’ view on the value of DHC despite the likelihood for functional deficit; that is, knowing the outcome, would they still chose to have the operation. In DESTINY II, 63% of surviving patients in the DHC group gave retrospective consent to treatment compared to 53% of those in the control group. Though, authors cautioned interpretation of these results, as 25 of 42 survivors were unable to answer due to severe aphasia or neuropsychological deficits^27^. An institutional study in Germany assessed 79 consecutive patients who underwent DHC for functional and psychological outcomes, as well as QoL and retrospective consent for the procedure^31^. Despite patients >60 years having worse functional outcomes after DHC, indicators of QoL and retrospective consent were higher than younger individuals. Of patients <60 years of age, 63% reported retrospective consent for DHC (29% declined), older patients reported 82% consent, and none declined. Older patients also reported higher scores for all items on the SF-36 questionnaire, with the exception of ‘General health’, and the use of antidepressants was significantly lower in the older group: 9 vs. 58% in younger patients^31^.

The aforementioned discrepancy between an individual’s report of a high QoL, despite serious disability that most external observers would view as a poor QoL, is termed the “disability paradox”^2^. In these situations, patient reported QoL measures are less dependent on physical ability, and are more reflective of one’s ability to sustain positive social relationships and engage in the external environment. Long-term consequences and QoL of both patients and their partners continue to be a point of investigation. The ongoing Restore4Stroke study is working to elucidate how QoL is impacted by health condition (pre-stroke and stroke-related health conditions), personal factors (coping and illness cognitions), and environmental factors (caregiver burden and social support)^41^. Overall, QoL measures and the prevalence of patients who retrospectively consentto DHC despite functional outcomes should be considered during counseling of patients and caregivers on postoperative expectations.

## Conclusion

While the utility of DHC in patients with malignant cerebral edema has been shown in patients <60 years of age, the role of surgery in older patients is more complex. Recent clinical trials, institutional studies, and population analyses suggest that surgical decompression in older patients is lifesaving, but often results in survival with moderate or severe disability. What constitutes an acceptable outcome for these patients is controversial, and expectations for post-operative quality of life should be thoroughly communicated to patients and caregivers before surgery, and shared decision-making with this information should occur when possible. This literature review underscores the inherent complexity in balancing scientific evidence, clinical expertise and patient preference when considering hemicraniectomy for space-occupying infarction in older adults.

## Figures and Tables

**Table 1.** Modified Rankin Scale (mRS). Scores are used to measure the degree of disability in patients who have had a stroke.

mRS Score	Score Description
0	No symptoms.
1	No significant disability. Able to carry out all usual activities, despite some symptoms.
2	Slight disability. Able to look after own affairs without assistance, but unable to carry out all previous activities.
3	Moderate disability. Requires some help, but able to walk unassisted.
4	Moderately severe disability. Unable to attend to own bodily needs without assistance, and unable to walk unassisted.
5	Severe disability. Requires constant nursing care and attention, bedridden, incontinent.
6	Dead

**Table 2.** Summary of key studies evaluating DHC in older patient populations

First Author, Publication Year, Location	Study Design Setting	Age Groups (Years)	Number of Patients	Key Findings
Carter, 1997 USA	Retrospective Academic institution	<50 ≥50	DHC 11	Of surviving patients, 5/5 patients <50 years old had good postoperative mobility and self-care (BI scores >60), verses 3/6 older patients.
Holtcamp, 2001 Germany	Retrospective Academic institution	>55	DHC12 Med 12	Of DHC patients, 8/12 survived. None of the survivors had a BI score above 60 or a mRS <4.
Walz, 2002 Germany	Retrospective Academic institution	<45 ≥45	DHC 18	Patients <45 years had a significantly better outcome than patients ≥45 by BI scores and survival rates.
Gupta, 2004 USA	Systematic review	<50 ≥50	DHC 138	Of 75 patients who were >50 years of age, 80% were dead or severely disabled compared with 32% of the 63 patients ≤50 years of age.
Uhl, 2004 Germany	Retrospective Academic institutions	<50 ≥50	DHC 188	Poor outcome (Glascow Outcome Score ≤3) was significantly associated with age >50 years.
Yao, 2005 China	Retrospective Academic institution	<60 ≥60	DHC 25	Mortality was 7.7% in younger patients (aged <60 years) compared with 33.3% in elderly patients (aged ≥60 years). Younger patients also had higher BI scores and were more likely to achieve mRS ≤3.
Curry, 2005 USA	Retrospective Academic institution	<40 ≥40	DHC 38	BI score and ability to walk were strongly correlated with age but not time to surgery, volume of infarction, or craniectomy size.
Rabinstein, 2006 USA	Retrospective Academic institutions	Range 15-73 Linear analysis	DHC 42	All but one of the patients with favorable recovery (mRS ≤3) were younger than 55 years. Older age was an independent predictor of poor outcome (OR 2.9 [95% CI: 1.04 to 8.07] per 10-year increase in age.
Zhao, 2012 China	RCT Multicenter trial	<60 ≥60	DHC 24 Med 23	For patients up to 80 years, DHC within 48 hours of stroke onset increases survival and likelihood of good functional outcome (mRS ≤3).
Tsai, 2012 China	Retrospective Military Hospital	<60 ≥60	DHC 37 Med 42	DHC improved survival of all age groups. There was no significant difference in functional outcome between patients <60 versus ≥60 years of age.
Yu, 2012 Korea	RCT Academic institution	<60 ≥60	DHC 58 Med 73	Age (≥70 years vs. < 70 years) did not statistically differ between groups for the six-month mortality rate.
Inamasu, 2013 Japan	Retrospective Academic institution	61-70 >70	DHC 18	30-day mortality rate was significantly higher in the group that was >70 years of age (0% vs 60%) than in the group that was 61 to 70 years of age.
Frank, 2014 North America	Randomized pilot study Multicenter trial	Range 18-75 Linear analysis	DHC 14 Med 10	HeADDFIRST: At 6 months, mortality rate for conservatively treated patients was 40%; DHC, 36%. Authors attributed relatively low mortality rate in conservative treatment group (compared to European RCTs) to (1) older patients having more brain atrophy and ability to tolerate cerebral edema better than younger patients, and (2) strict adherence to a standardized medical management protocol.
Juttler 2014 Germany	RCT Multicenter trial	>60	DHC 56 Med 56	DESTINY II: DHC improved outcomes compared to medical management: survival without severe disability (38% vs 18%, respectively); mRS=4 (32% and 15%); and mRS=5 (28% and 13%).
Suyama, 2014 Japan	Retrospective Multicenter survey	<60 ≥60	DHC 355	Of all DHC patients, 80.2% were ≥60 years of age. Age was not an independent predictor of mortality. At 3 months, only 5.2% of patients had mRS ≤3.
Lu, 2015 China	Meta analysis	<60 ≥60	DHC 747	DHC within 48 hours improved patient survival for all age groups. The proportion of older patients with poor functional outcome (88.3%) was significant higher than that of younger patients (66.8%).
Ragoschke-Schumm, 2015 Germany	Prospective database and interview, Academic institution	<60 ≥60	DHC 79	Despite impaired functional outcome after DHC, indicators of quality of life and retrospective consent are higher for patients older than 60 years over the long term.
van Middelaar, 2015 Netherlands	Systematic review	<60 ≥60	DHC 459	Patients <60 years old had a better functional outcomes (mRS ≤3) and reported quality of life (surveys) in comparison with older patients.
Dasenbrock, 2016 USA	Retrospective Nationwide database	<60 61-70 >70	DHC 1673	DHC associated with reduced mortality in all age groups. DHC patients >60 years experienced higher odds of mortality (32.4%), discharge to institutional care (47.1%), and a poor outcome (77.0%) compared with younger patients.
Fehnel 2016 USA	Retrospective Nationwide database	>65	DHC 130	There is a high rate of mortality among older stroke patients undergoing DHC. Most survivors of DHC are not permanently institutionalized (75% home at 1 year)

BI: Barthel Index; DHC: decompressive hemicraniectomy; Med: medical treatment; mRS, modified Rankin Scale; RCT: randomized controlled trial.

## Abbreviations

BI: Barthel Index
DHC: decompressive hemicraniectomy
EQ-5D: European Quality of Life Scale
mRS: modified Rankin Scale
QoL: quality of life
RCT: randomized controlled trial
